# The Bonding Mechanism of the Micro-Interface of Polymer Coated Steel

**DOI:** 10.3390/polym12123052

**Published:** 2020-12-19

**Authors:** Jiyang Liu, Qingdong Zhang, Boyang Zhang, Mingyang Yu

**Affiliations:** School of Mechanical Engineering, University of Science and Technology, Beijing 100083, China; zhangby@ustb.edu.cn (B.Z.); 15600990167@163.com (M.Y.)

**Keywords:** polymer coated steel, micro-interface, bonding mechanism, molecular dynamics (MD), quantum mechanics (QM)

## Abstract

As food and beverages require more and more green and safe packaging products, the emergence of polymer coated steel (PCS) has been promoted. PCS is a layered composite strip made of metal and polymer. To probe the bonding mechanism of PCS micro-interface, the substrate tin-free steel (TFS) was physically characterized by SEM and XPS, and cladding polyethylene terephthalate (PET) was simulated by first-principles methods of quantum mechanics (QM). We used COMPASS force field for molecular dynamics (MD) simulation. XPS pointed out that the element composition of TFS surface coating is Cr(OH)_3_, Cr_2_O_3_ and CrO_3_. The calculation results of MD and QM indicate that the chromium oxide and PET molecules compound in the form of acid-base interaction. The binding energies of Cr_2_O_3_ (110), (200), and (211) with PET molecules are −13.07 eV, −2.74 eV, and −2.37 eV, respectively. We established a Cr_2_O_3_ (200) model with different hydroxyl concentrations. It is proposed that the oxygen atom in C=O in the PET molecule combines with –OH on the surface of TFS to form a hydrogen bond. The binding energy of the PCS interface increases with the increase of the surface hydroxyl concentration of the TFS. It provides theoretical guidance and reference significance for the research on the bonding mechanism of PCS.

## 1. Introduction

Polymer coated steel (PCS) a layered metal polymer composite strip mainly used in the food packaging industry [1]. It has good mechanical properties, corrosion resistance, and longer preservation of food taste, and is widely used in high-grade beverage and food packaging. For food-grade packaging material, the technical requirements are increasing [2,3]. At present, Europe, the United States, Japan, South Korea, and other countries have carried out much research on PCS materials, including the PCS coating process [4,5], coating quality characterization [6,7], heat transfer during the coating process [8], PCS heat treatment [9], PCS application [10], film surface modification [11] and so on. In this paper, the micro-interface bonding mechanism of PCS is studied by the method of coating experiment combined with simulation. An effective method to improve the quality of the film is proposed. 

PCS materials were first developed and applied by Japan in the 1980s. Later, European countries and the United States also carried out related research. PCS is a metal-polymer composite material prepared by using chrome-plated steel strip as the base material and polymer film as the covering material through a roll-pressing hot-melt film process. Commonly used polymer films are polyethylene terephthalate (PET), polyethylene (PE), polypropylene (PP), etc., among which food-grade PET is the most common material [12,13]. 

The adhesion and mutual bonding of polymer materials on metal surfaces is an important research direction in the field of surface science [14,15,16].At present, there are four main types of metal and non-metal adhesion mechanisms generally recognized in the world: mechanical interlocking theory, electronic theory, diffusion theory, and adsorption theory [17]. 

Regarding the micro-combination mechanism of the PCS interface, most scholars in the world agree with the hydrogen bond combination theory proposed by Tanaka in 1987, which belongs to one of the above adsorption theories [18]. During the lamination process, the O in the carbonyl group in the polymer film combines with the H in the –OH on the TFS surface to form a hydrogen bond, but the theoretical and experimental basis for the formation of hydrogen bonds is not mentioned. Komai [19] pointed out that the hydrated chromium oxide on the surface of the TFS and the polymer film in the micro-interface of the PCS are connected in the form of hydrogen bonds. The hydrated chromium oxide acts as a proton acceptor to provide the –OH required to form hydrogen bonds. The proton donor provides –O. Zumelzu [20] proposed that the metal/polymer interface recombination mechanism can also be explained by another model, that is, the acid-base interaction mechanism, in which the oxygen atoms in the hydroxyl or benzene ring in PET share their electrons with chromium. In summary, there are two recombination mechanisms at the microscopic interface of PCS: hydrogen bond recombination and acid-base interaction mechanism. Previous studies have made important contributions to the study of the interface bonding mechanism of PCS. However, with the development of science and technology, the use of advanced experimental equipment and calculation methods to quantitatively describe the bonding process of the PCS micro-interface is a direction that still needs to be studied in the PCS field.

With the substantial improvement of computer performance, computer simulation technology has become a powerful tool for us to study the micro-scale of materials. According to previous research, the first principles of quantum mechanics (QM) and molecular dynamics (MD) have become advantageous tools for studying the interaction mechanism between metals and non-metals [21,22,23,24]. MD can simulate the interaction law between atoms in a defined complex system on the atomic scale [25,26,27,28]. QM can study the motion law of microscopic particles in the material world. Using first principles to study the principle of interaction between atomic nuclei and electronic components is an effective method. Seunghwa [29] used molecular dynamics (MD) simulation combined with finite element analysis (FEA) to study the mechanical behavior of oxygen-functionalized single-layer graphene/polyethylene (PE) nanocomposites. Ghasem Bahlakeh [30] used MD and QM methods to calculate the interfacial interaction mechanism between FeO, Fe_3_O_4_, and Fe_2_O_3_ and epoxy resin. By establishing and simulating the interaction law between the hydroxylated Fe_3_O_4_ surface and epoxy resin, it is proposed that increasing the concentration of hydroxyl on the surface of iron oxide can improve the interface bonding strength.

In this paper, from the experimental point of view, the chemical composition of the TFS surface coating on the coated iron substrate was measured by XPS, and then the modeling and simulation were carried out. The bonding process of PET and chromium oxide on the micro interface of PCS was studied by using the first principles method of molecular dynamics and quantum mechanics. In order to comprehensively evaluate the composite mechanism between chromium oxide and PET, three surface models of chromium oxide with different crystal face indexes were established, the binding energy between them and PET was calculated, and the binding modes of the three surfaces with PET were analyzed [31]. At the same time, the surface model of chromium oxide with different hydroxyl concentration was established to analyze the combination mode of hydroxyl and PET and the change rule of binding energy with the increase of hydroxyl concentration. The simulation results in this paper provide theoretical guidance for the study of the interface recombination mechanism of PCS. From the perspective of molecular dynamics and quantum mechanics, the hydrogen bonding and acid-base interaction between PET and TFS are described, which confirms and explains the hydrogen bond recombination theory and acid-base interaction theory proposed by predecessors. It provides a reference for the future research of PCS.

## 2. Experiment and Methods

### 2.1. Sample Preparation

**Figure 1:**
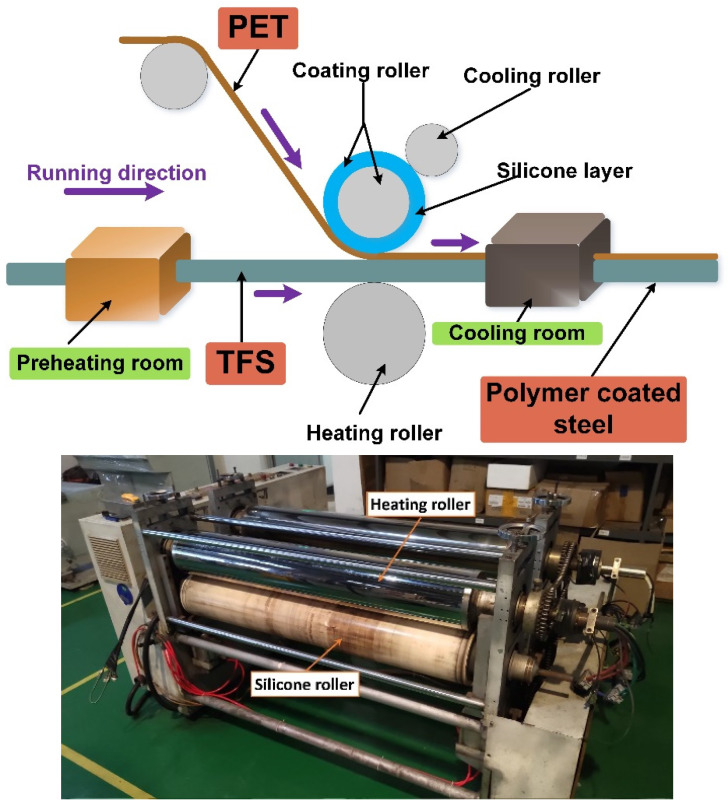
Equipment diagram and schematic diagram of the coating process.

**Figure 2:**
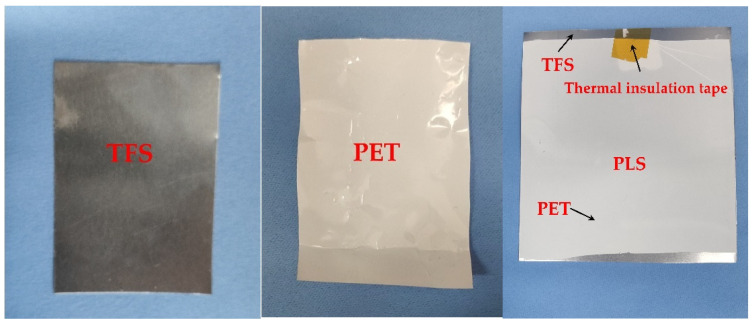
Raw materials and polymer coated steel (PCS) samples.

The PET film used in the preparation of PCS comes from the China Baowu Steel Group. The TFS comes from Shanghai Yigang Cangchu Co., Ltd. Laminating equipment (Laminator equipment, Shanghai Lianjing Co., Ltd., Shanghai, China) is used for the coating test of TFS and PET. The roll pressing and hot fusion coating process of PCS is shown in Figure 1. The PET film and TFS are coated under the double pressure of the heating roller and the silicone roller. During the laminating process, the heating roller acts as a heat source to continuously provide heat to the TFS. The TFS transfers the heat to the PET film and then to the silicone roller. The silicone roller maintains a constant temperature under the action of the cooling roller. Therefore, under the dual effects of the heating of the TFS and the cooling of the silicone roller, a temperature gradient occurs inside the PET along the thickness direction, forming a partial melting [32]. The PET film was completely melted on the side near the TFS, but not on the side near the silicone roller. After the PET film and TFS leave the heating roller and the silicone roller, the PET film is quickly cooled with a cooling liquid to quickly solidify the melted part of the PET to avoid recrystallization of the PET. The final preparation is the PCS material, as shown in Figure 2 [33]. Experimental conditions used for the film coating were a heating roller temperature of 500 K, plus coating speed of 15 m/min; the pressure roller is coated 1000 N.

### 2.2. XPS and SEM

**Figure 3:**
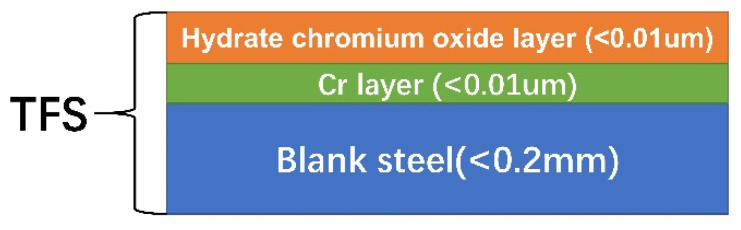
Schematic diagram of the tin free steel (TFS) surface coating structure.

The chemical elements and composition of the chromium plating layer on the surface of TFS was carried out using X-ray photoelectron spectroscopy (XPS, Thermo Fisher Scientific ESCALAB 250Xi, Waltham, MA, USA). The thickness of TFS is 0.18 mm, as shown in Figure 3. The test area of the sample is 10 mm × 10 mm. Firstly, the type and concentration of elements on the surface of the TFS are measured, and then the spectrum is fitted with XPSPEAK software to infer the main components of the coating on the surface of the TFS.

The morphology and microstructure of the TFS surface and blank steel was investigated by a scanning electron microscope (SEM-EDS, ZEISS Gemini 500, Carl Zeiss, Germany). The uniformity of the TFS surface coating was determined by comparing the SEM scan images of the TFS and blank steel. Line scan analysis of the two samples was performed using an EDS analyzer attached to the SEM. The thickness of the TFS and blank steel is approximately 0.18 mm and the size of the two samples is 10 mm × 10 mm.

### 2.3. MD and QM Simulation

#### 2.3.1. PET and the TFS Model

**Figure 4:**
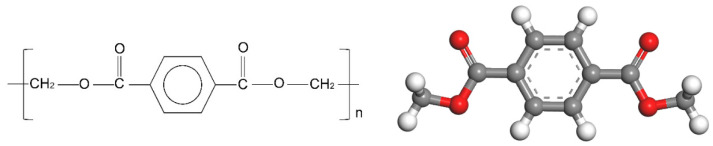
Polyethylene terephthalate (PET) molecular formula and model diagram (white is H atom, red is O atom, gray is C atom).

The polymer films commonly used in PCS materials mainly include polyethylene terephthalate (PET), polyethylene (PE), polypropylene (PP), etc., of which PET is the most common. In this paper, PET was selected as the coated film for research [34]. According to the principle of PCS coating, the PET film undergoes a transition from a highly elastic state to a viscous fluid state to a molten state under the action of heating on the surface of the substrate. Among them, the activity of the PET molecular segments within the material gradually increases with the increase in temperature, and the PET molecules in the molten state are combined with the surface of the substrate through hydrogen bonds [35]. According to the PET molecular formula, a PET molecular model was established using material studio software, as shown in Figure 4.

The substrate TFS used for PCS is prepared by simultaneously electroplating metallic chromium and chromium hydrated oxide on the surface of ordinary strip steel using the one-liquid method or two-liquid method [36,37]. Figure 3 shows the coating structure of the TFS surface. The surface of blank steel has a metal chromium layer with a thickness of less than 0.01um, and then there is a layer of hydrate chromium oxide layers with a thickness of less than 0.01um on the metal chromium layer. Therefore, the interface bonding mechanism of PCS is the combination of hydrogen chromium oxide layers and PET [1,20].

XPS results show that the main components of hydrogen chromium oxide layers are Cr_2_O_3_ and a large number of bound water molecules. Therefore, a Cr_2_O_3_ surface model with a (110), (200), and (211) crystal face index and Cr_2_O_3_ surface with different hydroxyl concentration were established, respectively. The Cr_2_O_3_ model is taken from the database in Materials Studio 8.0. The lattice constants [38,39] are a = b = 4.9589 Å, c = 13.59308 Å, α = β = 90°, γ = 120°, and the cleave surface tool is used to cut out Cr_2_O_3_ (110); the three structural models of Cr_2_O_3_ (200) and Cr_2_O_3_ (211) are respectively set at 13.156 Å, 21.355 Å, and 13.79 Å in thickness. 

**Figure 5:**
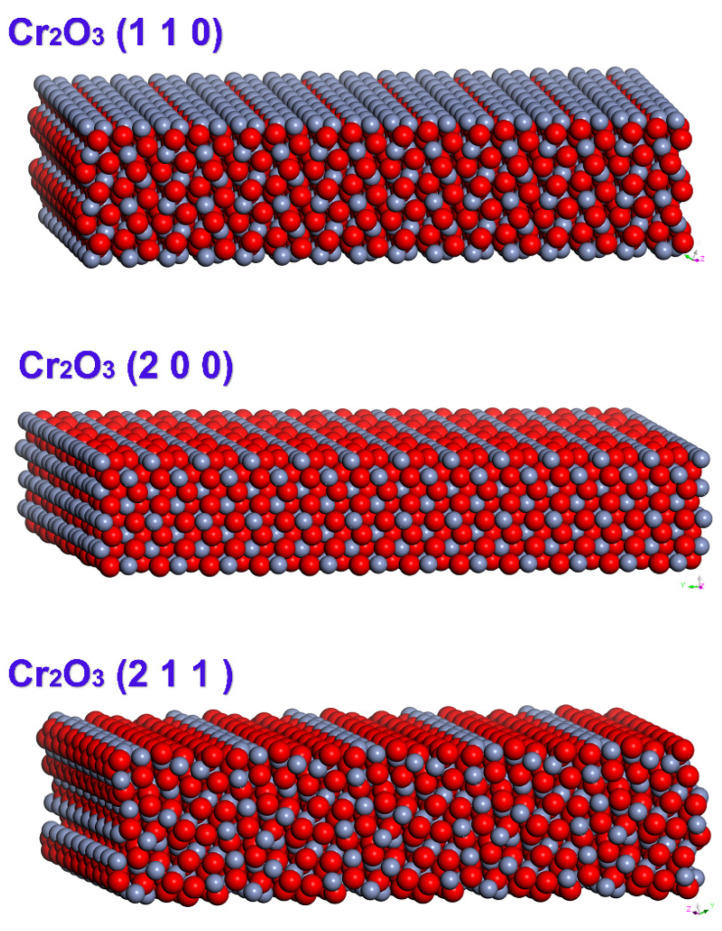
Cr_2_O_3_ surface model diagram (red is O atom, gray is Cr atom).

In order to more accurately simulate the interaction between the chromium oxide and PET molecular chains, the three models generated were enlarged in parallel to the x and y directions, and the three models used periodic boundaries in the three coordinate axis directions. In order to ensure that the PET molecules only interact with the upper surface of chromium oxide, a 50 Å vacuum layer is placed vertically above the three upper surfaces [40]. As shown in Figure 5, the final dimensions of the Cr_2_O_3_ (110), Cr_2_O_3_ (200), and Cr_2_O_3_ (211) models are 58.42 × 53.60 × 63.36, 59.51 × 54.37 × 60.17, and 56.37 × 53.60 × 62.82.

#### 2.3.2. MD and QM Simulation

Before calculating the model, it is necessary to optimize the structure of the PET molecular model to obtain the equilibrium structure of the PET molecular model, and use the first-principle electronic structure calculation of quantum mechanics to geometrically optimize the PET molecular structure. The simulation calculation is performed using the DMOL3 module in the Materials Studio 8.0 software, and the Perdew−Burke−Ernzerhof (PBE) functional in the generalized gradient approximation (GGA) proposed by Perdew is used to describe the exchange-correlation interaction between electrons [41]. The convergence accuracy is 1.0 × 10^−5^ eV/atom, the maximum force convergence accuracy acting on a single atom is 0.002 eV/ Å, the maximum pressure convergence accuracy is 0.1 GPa, and the maximum displacement convergence accuracy is 0.005 Å. The self-consistent accuracy of self-consistent filed (SCF) is set as the single atom energy converges to 1.0 × 10^−6^ eV, and the grid size of the k-point in the Brillouin zone is 1 × 1× 1.

We optimized the structure of the model system containing PET molecules and oxide surfaces, and obtain a balanced structure model with minimized energy. The model was subjected to 200ps MD simulation, and the simulation was performed under the condition of 500K temperature of the NVT system to obtain a balanced structure. Using the COMPASS force field in atomic simulation to simulate the interaction between PET molecules and chromium oxide molecules [42,43], Ghasem Bahlakeh [44] used the same method to study the interaction between epoxy resin and iron oxide molecular systems. Ewald- and atom-based methods were used to calculate the electrostatic interaction force and van der Waals force. The MD simulation used the Anderson monitor to monitor the temperature, and used the velocity calculus method to solve the Newtonian equation of motion, with an integration step of 1 ps. In the simulation process, all atoms in the oxide system are constrained, and the PET molecules do not impose constraints on the MD simulation.

#### 2.3.3. Interaction Energy Calculation

In order to quantitatively study the interaction mechanism between the micro-interfaces of the coated iron and comprehensively evaluate the binding ability between the surface molecules of chromium oxide and the PET molecular chains on a micro-scale, the binding energies between the PET material and the three crystal plane structure models were calculated. Using the equilibrium structure model obtained at the end of the MD simulation, the interface interaction energy between the PET molecular system and the chromium oxide surface model with three different crystal surface structures is calculated according to the following formula [42,43,44]:

(1) $$E=E_{total}-\left(E_{surface}+E_{PET}\right)$$

In the formula: E represents the binding energy between the PET molecular system and the oxide model; $E_{total}$ represents the total energy of the equilibrium structure after the MD simulation calculation; $E_{surface}$ represents the energy of the oxide surface; and $E_{PET}$ represents the energy of the PET molecule.

When calculating the total energy of the equilibrium structure after the MD simulation, we canceled all fixed atoms in the model to obtain an accurate total energy of the flat energy structure, and also canceled the constraints of all atoms during calculation.

## 3. Results and Discussion

### 3.1. Analysis of the Experimental Results

**Figure 6:**
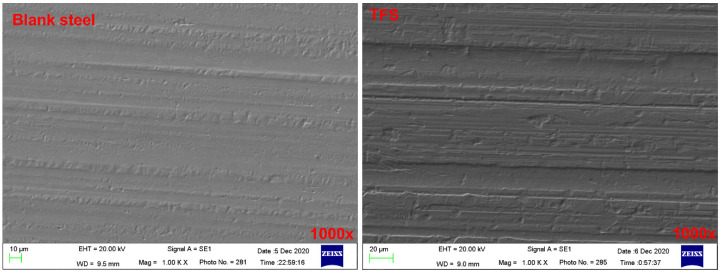
The SEM images of blank steel and TFS.

The microstructure of tin free steel (TFS) was evaluated by SEM analyses, as shown in Figure 6. By comparing the microscopic images of the blank steel plate and the chrome plate under different magnification conditions, it can be clearly seen that there are obvious grinding streaks on the surface of the blank steel plate, while the grinding streaks on the surface of the chrome steel plate are relatively inconspicuous. This is because the presence of the chromium-plated layer on the surface of the chrome-plated steel sheet weakens the microscopic morphology of the surface of the blank steel sheet.

**Figure 7:**
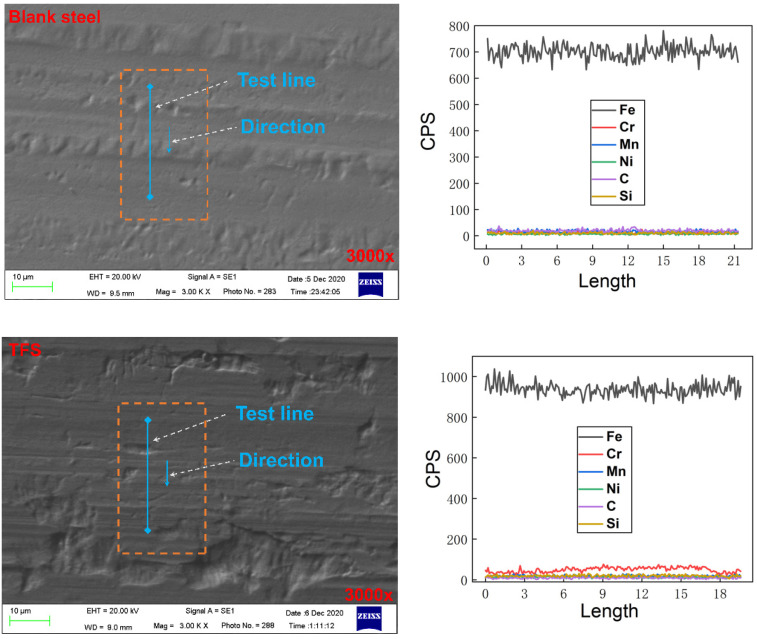
The SEM images and EDS curve of the blank steel and TFS.

Figure 7 shows the SEM image of blank steel and TFS samples under 3000 times. We randomly selected a test line in the image for EDS scanning. It was found that the Cr element on the surface of the TFS is significantly higher than other elements such as Mn, Si, C, and Ni, while the content of the Cr element on the blank steel surface is very close to other elements. This shows that the chromium layer on the TFS surface completely covers the blank steel surface. It further illustrates that the bonding between the PCS interface occurs between the plating layer and the PET.

**Figure 8:**
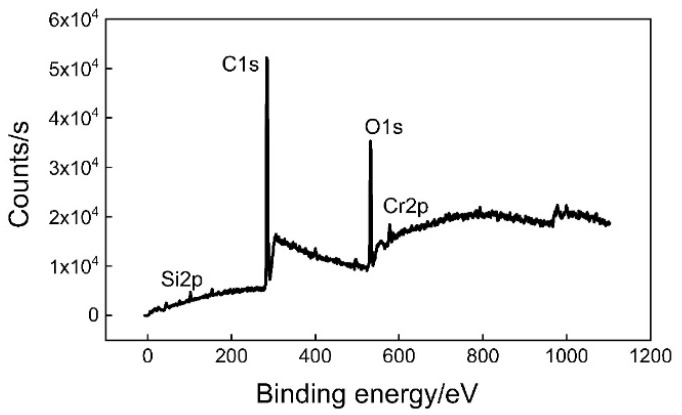
XPS spectra of the TFS surface.

The chemical composition of chromium coating on the TFS surface was analyzed by XPS. It can be seen from Figure 8 that there are signal peaks of Cr2p, O1s, Si2p, and C1s on the surface of the TFS. Among them, the C element is the result of introducing contaminant elements in the sample analysis process, and there is no C element on the surface of the coating. Part of the O element must come from the oxide or hydroxide formed by Cr and O, but from the content of the surface elements in Table 1, the content of oxygen is much greater than that of pure Cr oxide. It is likely that part of it comes from bound water molecules. The Si element comes from additives in the Cr plating process.

**Figure 9:**
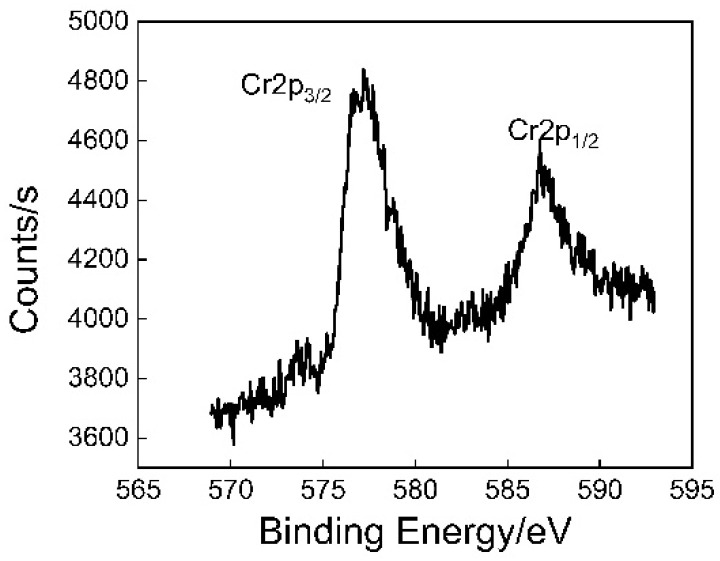
The XPS spectra of Cr in the TFS.

Figure 9 shows the spectrum of Cr on the TFS surface. It can be seen that the chemical shift of the Cr moves towards higher binding energy. This indicates that chromium compounds exist on the surface of TFS. Both Cr2p1/2 and Cr2p2/3 have broad peaks, indicating that chromium may have multiple valence states or molecular structures. 

**Figure 10:**
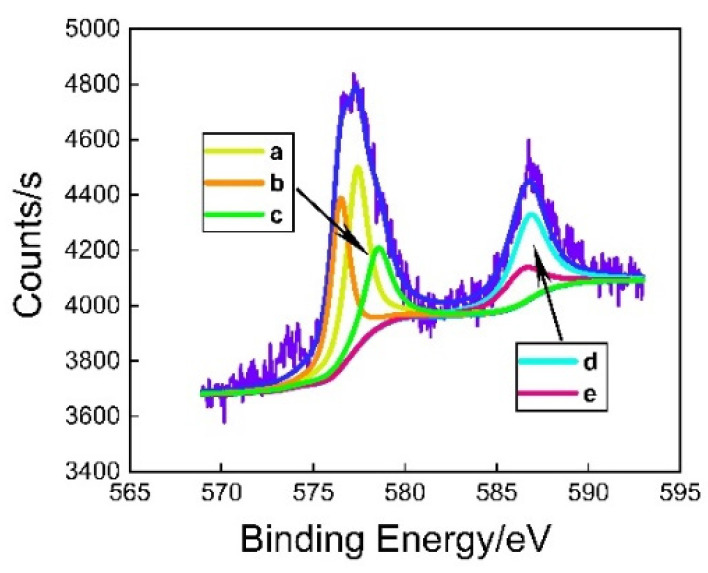
The fitting curve of the Cr XPS in the TFS.

We used XPSPEAK software to fit the peaks of Cr2p3/2 and Cr2p1/2, as shown in Figure 10. Cr2p3/2 fits three sub-peaks, a, b, and c, and the corresponding binding energies are 577.4, 576.5, and 578.5 eV, respectively. We found the binding energy data in national institute of standards and technology (NIST), and the corresponding binding energy compounds are Cr(OH)_3_, Cr_2_O_3_, and CrO_3_. Cr2p1/2 fit two sub-peaks, d and e, and the corresponding binding energies are 586.4 and 586.8 eV, respectively. We found the binding energy data in NIST. The corresponding binding energy compounds are Cr_2_O_3_ and Cr(OH)_3_. Combined with the fitting results of Cr2p3/2 and Cr2p1/2, the chemicals of the surface coating of TFS are mainly Cr(OH)_3_, Cr_2_O_3_, and CrO_3_, as shown in Table 2. The TFS is prepared by electroplating the hydrated chromium oxide layer on the surface of blank steel by the method of cathodic plating. The electrolyte used is a CrO_3_ solution, so the CrO_3_ in the coating should be the residue of the electrolyte on the surface of the steel sheet. Cr(OH)_3_ and Cr_2_O_3_, are the products generated during the electroplating process.

Since the coating process of PCS is a roll composite at high temperature, Cr(OH)_3_ and Cr_2_O_3_ can react to produce Cr_2_O_3_ at a high temperature. Therefore, the uppermost compound on the TFS surface during coating is Cr_2_O_3_ and bound water molecules.

### 3.2. Interface Bonding Mechanism

#### 3.2.1. MD and QM Simulation Results

**Figure 11:**
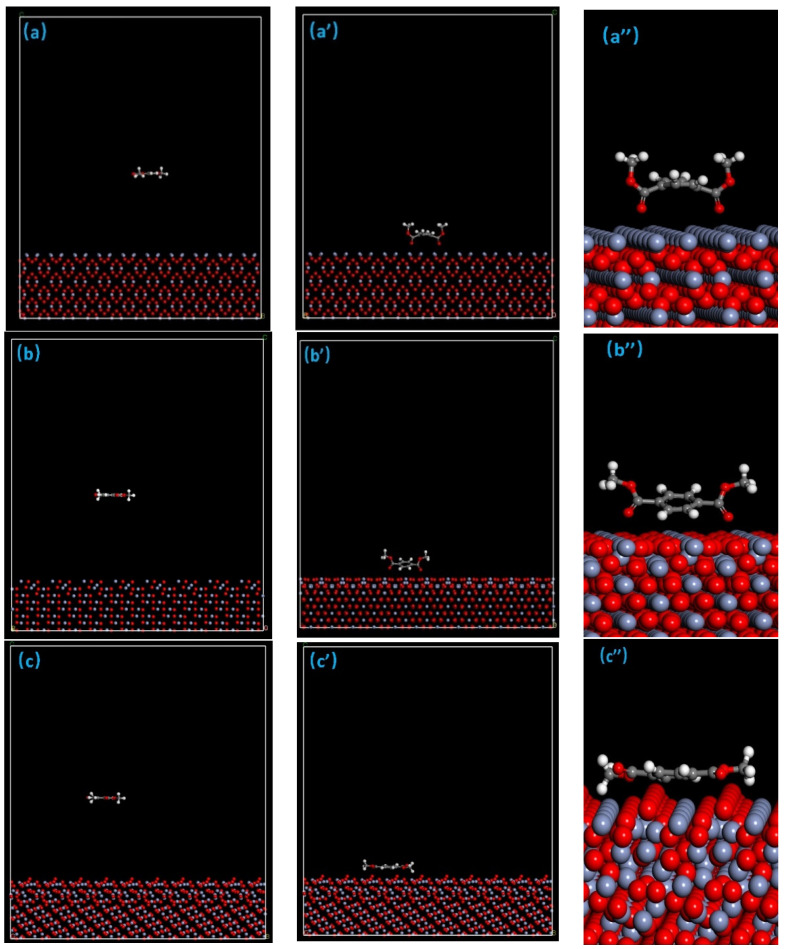
The initial and final configurations of the Cr_2_O_3_ (110) (**a**,**a′**,**a″**) surface, Cr_2_O_3_ (200) (**b**,**b′**,**b″**) surface and Cr_2_O_3_ (211) (**c**,**c′**,**c″**) surface interacting with PET molecules (white is H atom, red is O atom, gray is C atom).

The MD simulation calculation was carried out using the modeling method described in Section 2.3.2. Figure 11 indicates the initial input cells and the corresponding final configurations obtained at the end of 200 ps MD simulations for PET molecules on the Cr_2_O_3_ (110) (a, a′) surface, Cr_2_O_3_ (200) (b, b′) surface, and Cr_2_O_3_ (211) (c, c′) surface. Comparing Figure 11a and Figure 11a′, it can be found that after a certain period of MD simulation, the distance between the PET molecule and the highest atomic layer on the Cr_2_O_3_ (110) surface is greatly reduced, and the PET molecules move to the surface of Cr_2_O_3_ (110). This indicates that the PET molecules have compounded with the Cr_2_O_3_ (110) surface. Observing the final structure after MD simulation, it is found that the oxygen atom in the PET molecule C=O bond is the closest to the highest atomic layer on the Cr_2_O_3_ (110) surface, similar to the oxygen atom in the PET molecule C=O bond pulling the entire PET molecules to the surface of chromium oxide, which is caused by the Lewis acid-base interaction between the electrons in the O atoms and the surface of positively charged Cr ions. O atoms act as electron donors to provide electrons to the surface of the chromium oxide and are positively charged. The Cr ions on the metal surface accept electrons as electron acceptors to make O atoms move to the surface of the chromium oxide, which explains the micro-interface recombination mechanism of coated iron, that is, the coated iron is formed through the oxygen atom in the C=O bond of the PET molecule and Cr_2_O_3_ (110). The compound was formed by the Lewis acid-base interaction between the Cr atoms on the surface.

Figure 11b,b′,b″ and Figure 11c,c′,c″ are the initial structure models of PET and Cr_2_O_3_ (200), and the Cr_2_O_3_ (211) plane and the final structure model after 200ps MD simulation. Combining Figure 11a,a′,a″ by comparing the initial structure and final structure of the three groups of models, it can be found that the PET molecules in the three groups of models have all moved to the surface of the chromium oxide, and the same final result is obtained after a longer MD simulation. In the structural model, the positioning of PET molecules near the surface of the chromium oxide proves that the PET molecules are compounded with the surface of the chromium oxide. Comparing Figure 11b′,c′, it can be found that the PET molecular shapes in the final structural models of the two crystal planes are relatively similar, which is different from that in Figure 11a′, where the oxygen atoms in PET are the closest to the highest atomic layer of chromium oxide. 

Figure 11b′,c′ show that the H atom in the benzene ring is the closest to the highest atomic layer of chromium oxide, while the O in C=O is next to the highest atomic layer of chromium oxide. This is caused by the different structure of the chromium oxide surface, Cr_2_O_3_ (110). The highest layer atoms on the surface are all Cr atoms, while the highest layer atoms on the Cr_2_O_3_ (200) surface are both Cr atoms and O atoms, coexisting. Although all the highest layer atoms on the Cr_2_O_3_ (211) surface are all Cr atoms, the distance between the Cr atoms in each row is wide. The O atoms under the chromium layer are not completely covered by chromium atoms. From Figure 11b′,c′, can be seen that the H atom on the benzene ring in the PET molecule and the O on the surface of the chromium oxide are combined by forming a hydrogen bond, and the O in the C=O bond also occurs simultaneously with the surface Cr atom acid-base interaction.

#### 3.2.2. Interaction Energy Calculation

**Figure 12:**
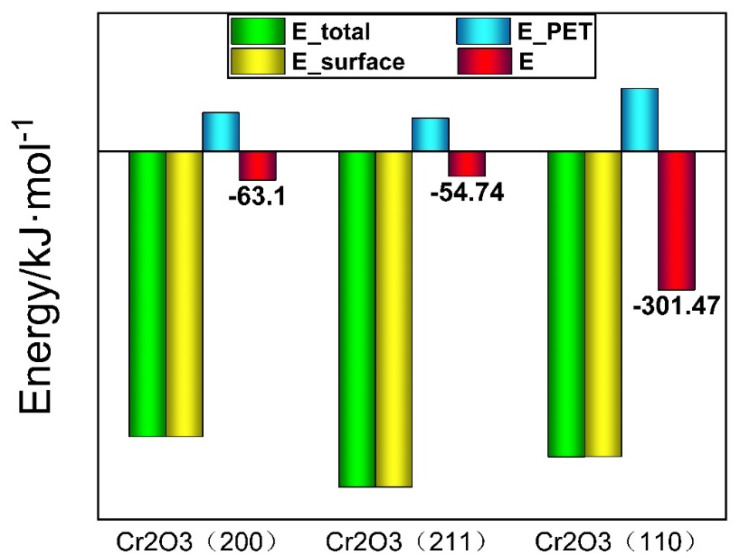
The binding energy of the Cr_2_O_3_ surface with PET.

According to the binding energy calculation formula in Section 2.3.3, the binding energy between the three model PET molecules and the chromium oxide surface was calculated. Before calculation, all atoms in the model were unconstrained. The calculation results are shown in Figure 12 and Table 3.

It can be seen from Table 3 that the binding energies between the surface of the Cr_2_O_3_ (110), (200), (211) and PET molecules are −301.47, −63.10, and −54.74 kJ/mol, respectively. Previous studies have shown that when the binding energy is −14.28 kJ/mol < E < 0 kJ/mol, it is physical adsorption; when the binding energy E < −14.28 kJ/mol, it is chemical adsorption. It can be seen that the composite type between the chromium oxide surface and the PET molecule is chemical adsorption, which further proves that the PET molecule and the chromium oxide surface are composited, and the binding energy between the three crystal plane structures of chromium oxide and the PET molecule is Cr_2_O_3_ (110) > Cr_2_O_3_ (200) > Cr_2_O_3_ (211).

#### 3.2.3. Radial Element Concentration Distribution

In order to further quantitatively analyze the mechanism of the combination of PET molecules and the surface of chromium oxide, the element radial concentration distributions of oxygen atoms and hydrogen atoms in the initial and final structures of the three models were calculated. Figure 12 shows the concentration distribution of oxygen atoms and hydrogen atoms in the initial and final structures of different crystal plane models along the direction perpendicular to the surface.

**Figure 13:**
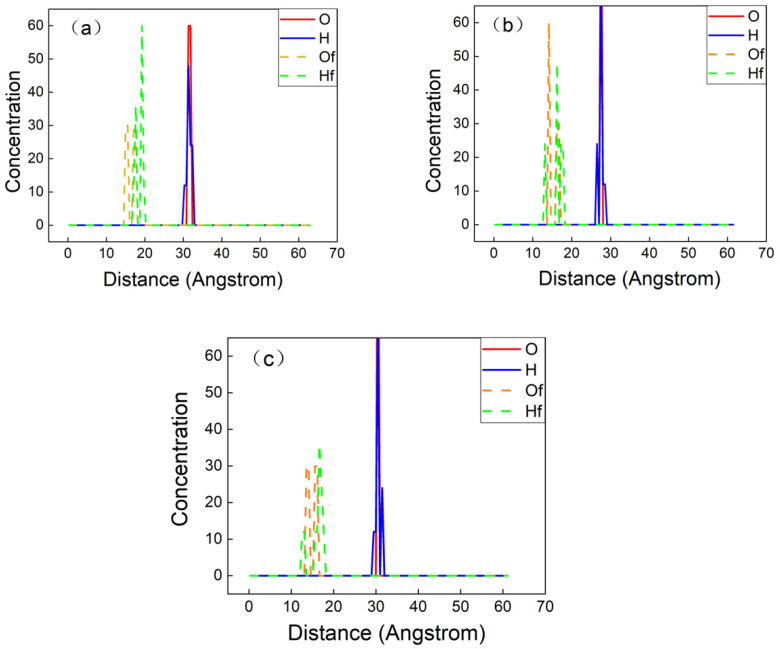
Concentration profiles of oxygen (O) and hydrogen (H) atoms in PET on top of (**a**) Cr_2_O_3_ (110), (**b**) Cr_2_O_3_ (200), and (**c**) Cr_2_O_3_ (211) surfaces before (O and H) and after (Of and Hf) MD simulations.

It can be seen from Figure 13 that the distance between the oxygen atoms and hydrogen atoms in the initial structure of the three models relative to the highest layer of the chromium oxide surface is approximately 30 Å, and the distance between the oxygen atoms and carbon atoms in the final structure relative to the chromium oxide surface approximately 15 Å. It is further confirmed that the PET molecules have been composited with the surface of chromium oxide, and can be composited with the surface of chromium oxide with different crystal plane structures.

**Figure 14:**
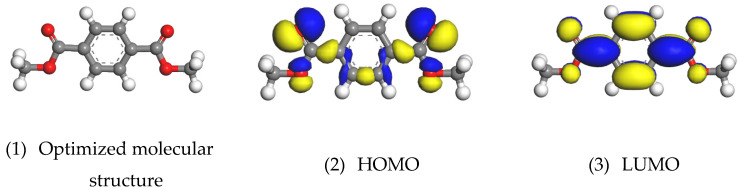
GGA/PW91-optimized structure and highest occupied molecular orbitals (HOMOs) and lowest occupied molecular orbital (LUMOs) of PET molecules.

The frontier molecular orbital theory (FMO) was used to further reveal the bonding mechanism between the PET film and the chromium oxide layer proposed by Zumelzu [20] through acid-base interaction. Figure 14 shows the global minimum energy structure of PET molecules, the respective distributions of the highest occupied molecular orbital (HOMO), and the lowest occupied molecular orbital (LUMO). HOMO orbitals are related to the active sites that have the greatest ability to interact with metal atoms and provide electrons to the empty electron orbitals on the surface of metal atoms. The LUMO orbital of the PET film represents the active site that accepts electrons from the filled orbital of atoms on the metal surface. 

It can be seen that the HOMO orbitals of the PET molecules are mainly oxygen atoms. The LUMO orbital is mainly a benzene ring and the carbon atom is connected to the benzene ring. The PET molecules donate delocalized electrons near the oxygen atoms to the low-energy unoccupied orbitals of the chromium atoms on the surface of the chromium oxide. PET molecules provide electrons, and the surface of the chromium oxide absorbs electrons, and recombines through acid-base interactions. 

**Figure 15:**
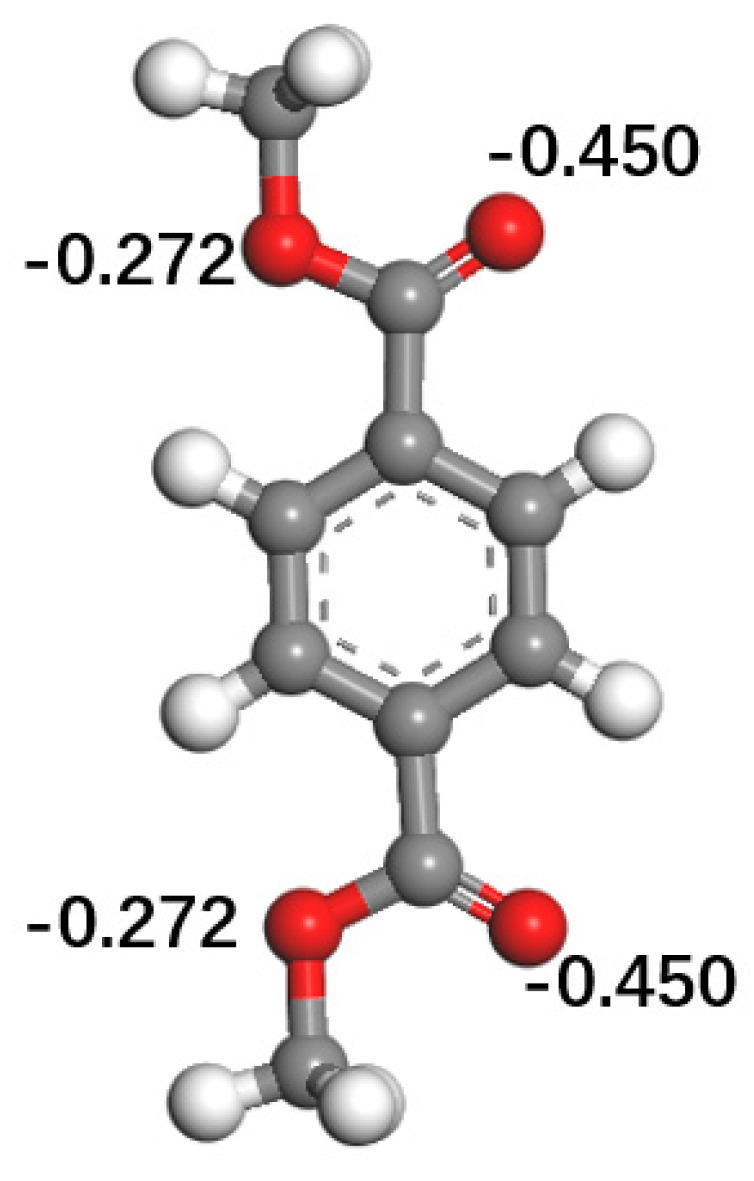
Partial atomic charge of PET molecules.

Figure 15 shows the charge of the oxygen atoms in the PET molecule. The charge of the oxygen atom in the C=O bond is −0.450, which is the highest electronegativity in the entire PET molecule. Therefore, the combination of the PET molecule and the chromium oxide molecule is mainly based on the O atom in the C=O bond. The final stable structure model of the three crystal planes in Figure 9 further illustrates that the oxygen atoms in the PET molecule and the chromium atoms on the chromium oxide surface are compounded through acid-base interactions.

### 3.3. Chromium Oxide Surface with Different Hydroxyl Content

**Figure 16:**
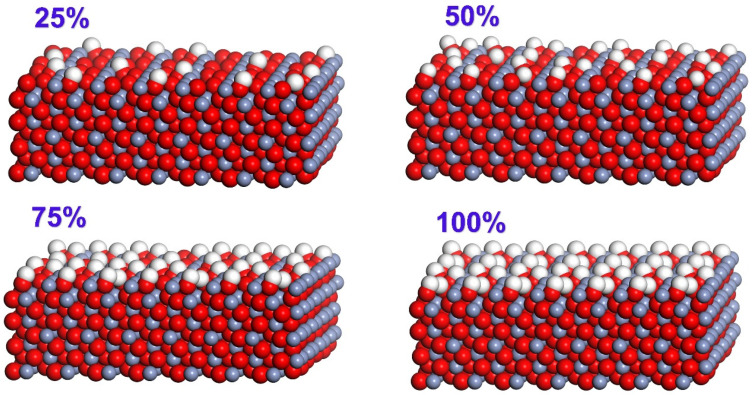
Cr_2_O_3_ (200) surface with different hydroxyl concentration (white is H atom, red is O atom, gray is C atom).

The XRD experiment results show that there are a large number of hydrogen and oxygen atoms on the surface of TFS strip steel. Other literature about layers has pointed out through experiments and analysis of the binding energy of chromium oxide that there are a large number of hydroxyl groups on the surface of TFS steel plate (quoted from Wuxi steel plate). Since all the atoms on the top layer of the Cr_2_O_3_ (110) are Cr atoms, the Cr_2_O_3_ (200) surface with a slightly larger binding energy is used as the base material model for modeling. Using the method of adding hydrogen atoms to the surface of oxygen atoms to build a hydroxylated surface model, we established 25%, 50%, 75%, and 100% hydroxyl concentration Cr_2_O_3_ (200) surface models, respectively. We placed the PET molecules in parallel at 50A above the Cr_2_O_3_ (200) surface with different hydroxyl concentrations to eliminate the effect of the position of the PET above the chromium oxide surface on the settlement result, as shown in Figure 16. The formation of hydrogen bonds adopts the definition method proposed by Ghasem. Ghasem defined two necessary conditions for the formation of hydrogen bonds when studying the interface bonding mechanism between different iron oxide types and epoxy resins: two hydrogen donor atoms (D) and hydrogen atoms (A) sharing one hydrogen atom (H) form a hydrogen bond when the following geometric conditions are met, where (1) the distance H··A should be less than 2.5A; and (2) the angle D−H··A should be greater than 90° [44].

**Figure 17:**
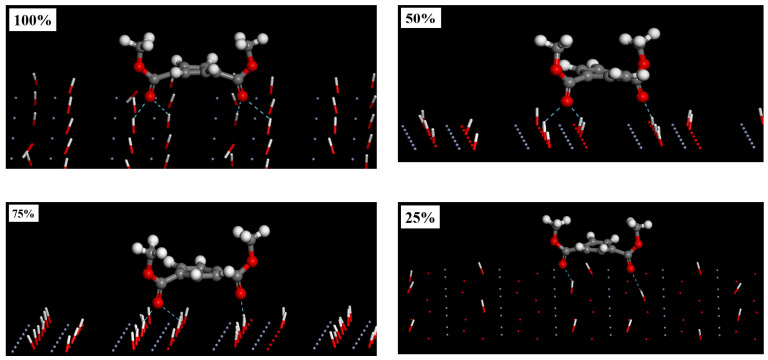
Side view of PET molecule complexation with Cr_2_O_3_ with different hydroxyl concentration (the cyan dotted line represents the hydrogen bond).

It can be seen from Figure 16 that the PET molecules in all models have moved to the surface. The oxygen atom in C=0 in the PET molecule migrates to the surface of the chromium oxide with the entire PET molecule, and recombines with the hydroxyl group on the surface of the chromium oxide to form a hydrogen bond. The cyan dotted line in Figure 17 represents hydrogen bonding. Corresponding to 25%, 25%, 25%, and 25%, the number of hydrogen bonds generated by the hydroxyl concentration model are 2, 3, 3, and 4, respectively. With the increase of hydroxyl concentration on the surface of chromium oxide, the number of hydrogen bonds gradually increases. 

**Figure 18:**
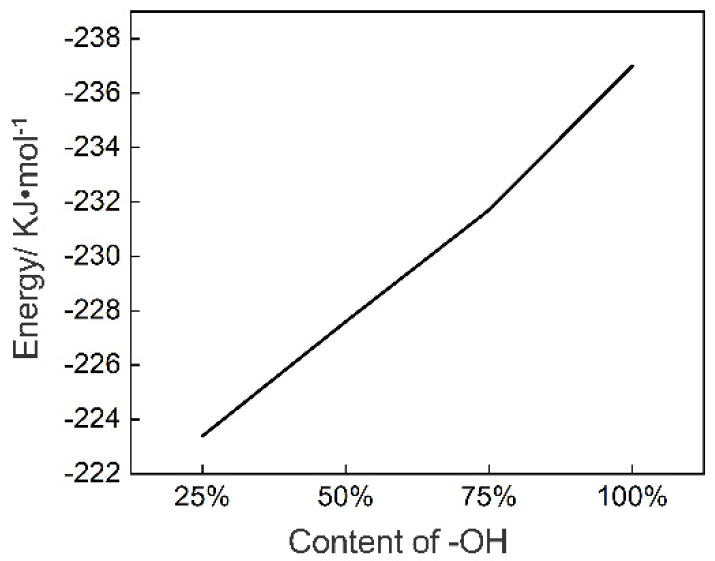
Different concentrations of hydroxyl groups.

We calculated the binding energy of the model using the formula in Section 2.3.3. The binding energies corresponding to 25%, 25%, 25% and 25% hydroxyl concentration models are −223.4, −227.6, −231.7, and −237 KJ/mol, respectively, as shown in Figure 18. With the increase of hydroxyl concentration on the surface of the chromium oxide, the binding energy between the PET and Cr_2_O_3_ (200) gradually increases, as shown in Figure 17. This is precisely due to the increase in the number of generated hydrogen bonds, which leads to the increase in binding energy.

## 4. Conclusions

The micro morphology of the coating on the surface of TFS was measured by SEM. The chrome-plated layer evenly covers the surface of the blank steel, and the presence of the chrome-plated layer weakens the grinding streaks on the surface of the blank steel. The chemical composition and full spectrum of the chromium plating layer on the surface of the TFS were obtained through XPS experiments. Using XPSPEAK software to fit the spectra, it is concluded that the composition of the chromium plating layer on the TFS surface is Cr(OH)_3_, Cr_2_O_3_, and CrO_3_.

The bonding mechanism of PCS micro-interface is explained by MD calculation combined with the first principles method. The chromium oxide surface and PET molecular models at the micro-scale were established respectively, and MD and QM simulations under certain parameter conditions were carried out.

The composite models of the Cr_2_O_3_ (110), Cr_2_O_3_ (200), and Cr_2_O_3_ (211) surfaces and PET molecules were established respectively. It is pointed out that PET molecules and chromium oxide molecules are compounded in the form of acid-base interactions. The carbonyl oxygen (electron pair donor = base) shares its electrons with chromium (electron pair acceptor = acid). We verified and proved the acid-base interaction theory proposed by Zumlzu [20]. The binding energy between Cr_2_O_3_ (110), Cr_2_O_3_ (200), Cr_2_O_3_ (211), and the PET molecules is −301.47 KJ/mol, −63.10 KJ/mol, and −54.74 KJ/mol. The binding energy between Cr_2_O_3_ (110) and the PET molecules is the largest.

A surface model of Cr_2_O_3_ (200) with different hydroxyl concentrations was established. Through MD and QM calculations, radial element concentration analysis, and frontier orbital theory, it is proved that the PET and hydroxylated chromium oxide surface are compounded in the form of hydrogen bonds. The oxygen atom in the C=O bond in the PET molecule combines with the hydroxyl group on the surface of Cr_2_O_3_ to form a hydrogen bond, which verifies the hydrogen bond recombination theory proposed by Tanaka [18]. It is calculated that the binding energies of the composite model with 25%, 50%, 75%, and 100% hydroxyl concentration are −223.4, −227.6, −231.7, and −237 KJ/mol, respectively. The increase in the concentration of hydroxyl on the surface of the chromium oxide helps to improve the interfacial binding energy. The key to improve the binding energy of the PCS interface is to increase the concentration of hydroxyl groups on the surface of TFS. Increasing the hydroxyl content in the hydrated chromium oxide layer on the surface of TFS is an effective way to improve the bonding strength of PCS.

## Figures and Tables

**Table 1:** The element content of the TFS surface.

Name	Peak BE	Atomic %
Cr2p	577.07	0.76
C1s	284.82	83.52
O1s	532.09	13.72

**Table 2:** The curve fitted by XPSPEAK corresponds to the compound in NIST.

Name	Number	Binding Energies/eV	Compounds
Cr2p3/2	b	577.4	Cr(OH)_3_
Cr2p3/2	a	576.5	Cr_2_O_3_
Cr2p3/2	c	578.5	CrO_3_
Cr2p1/2	e	586.4	Cr_2_O_3_
Cr2p1/2	d	586.8	Cr(OH)_3_

**Table 3:** The element content of the TFS surface.

	$E_{total}$ (kJ/mol)	$E_{surface}$ (kJ/mol)	$E_{PET}$ (kJ/mol)	E (kJ/mol)
Cr_2_O_3_ (200)	−620596.21	−620616.96	83.86	−63.10
Cr_2_O_3_ (211)	−729577.13	−729594.39	72.00	−54.74
Cr_2_O_3_ (110)	−663991.26	−663826.00	136.21	−301.47
